# Two Cases of Strictures after Percutaneous CT-Guided Radiofrequency Ablation for Renal Cell Carcinoma

**DOI:** 10.1155/2020/1205032

**Published:** 2020-01-13

**Authors:** Ortwin Heißler, Stephan Seklehner, Maximilian Fingernagel, Paul F. Engelhardt, Claus Riedl

**Affiliations:** ^1^Department of Urology, Landesklinikum Baden-Mödling, Baden, Austria; ^2^Paracelsus Medical University, Salzburg, Austria

## Abstract

Percutaneous radiofrequency ablation is a safe and effective minimally invasive treatment option in selected patients with T1a tumors of the kidney with a low complication rate. We describe two cases that developed the rare but severe complication of thermal injury-induced strictures of the upper urinary tract and its consecutive management.

## 1. Introduction

Percutaneous radiofrequency ablation (RFA) has proven to be a safe and effective treatment option for small renal masses (T1a) in selected patients [1]. The European Association of Urology (EAU) 2018 guidelines list RFA as an alternative treatment option to nephron-sparing surgery [2].

RFA induces thermal destruction of tumor cells at temperatures of approximately 100°C. Tumors are ablated by convective heat in concentric spheric zones around the RFA needle, so the risk of nontarget thermal damage of adjacent organs, e.g., bowel and ureter, needs to be taken into consideration. Pyelocalyceal injuries leading to stricture formation or ureteric strictures that require surgical, endoscopic, or radiological intervention are rare with a percentage of up to 4% [3].

We report the cases of two patients who developed asymptomatic hydronephrosis and kidney function deterioration as a result of stricture formation induced by nontarget thermal injury and its management.

## 2. Case Report One

**Figure 1:**
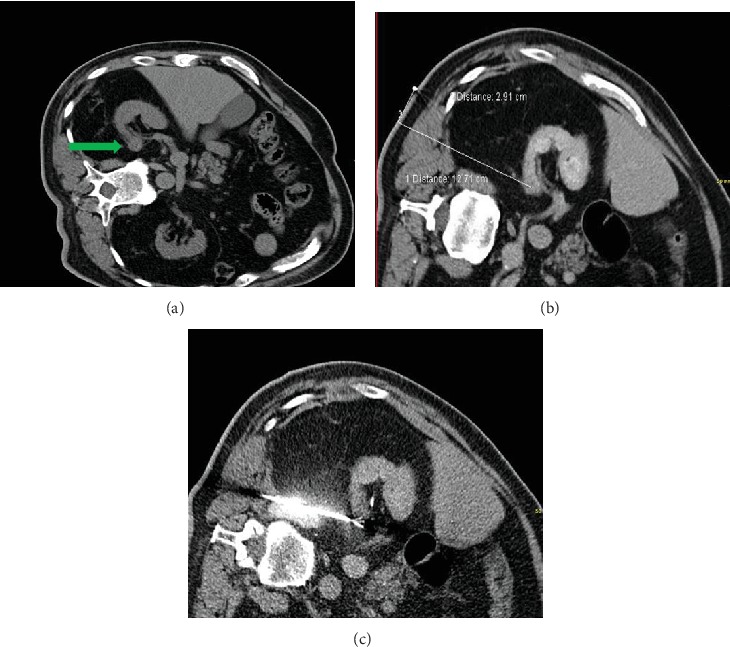
(a) 18 mm medially located tumor of the right kidney. (b) Planning the optimal ablation needle insertion path. (c) Placing the needle electrode into the tumor under image guidance.

A 70-year-old man underwent percutaneous RFA of an 18 mm renal tumor (Figure 1), after detailed explanation about risks, benefits, and the possibility of active surveillance, which he refused. The tumor was located medially in a duplex kidney with a ureter fissus to the level of lumbar vertebral body III. Biopsy was taken prior to RFA, showing a papillary renal cell carcinoma (RCC), Type 1, Fuhrman nuclear grade 1.

Since the patient wanted active treatment, percutaneous RFA was suggested because of significant comorbidities (coronary heart disease with a history of myocardial infarction and implantation of two coronary stents, arterial hypertension, and an increased body mass index) classified as ASA III.

In December 2016, a percutaneous CT-guided RFA (Modell 1500 RF Generator, 25 cm StarBurst XL Semi-Flex RFA Device, AngioDynamics, Queensbury, NY, USA) with two overlapping ablation zones, each 4 cm in diameter, with an average ablation temperature of 105°C and an ablation duration of eight and ten minutes was performed (Figures 1(b) and 1(c)). A final CT scan, excluding postinterventional urinoma, perirenal bleeding, hydronephrosis, and pneumothorax, was performed, and the patient was discharged on the first postinterventional day.

After four months, he was referred by his urologist because of an asymptomatic de novo hydronephrosis of the upper as well as the lower part of the duplex kidney and elevated serum creatinine that had increased from 1.3 mg/dl before RFA to 2.1 mg/dl.

**Figure 2:**
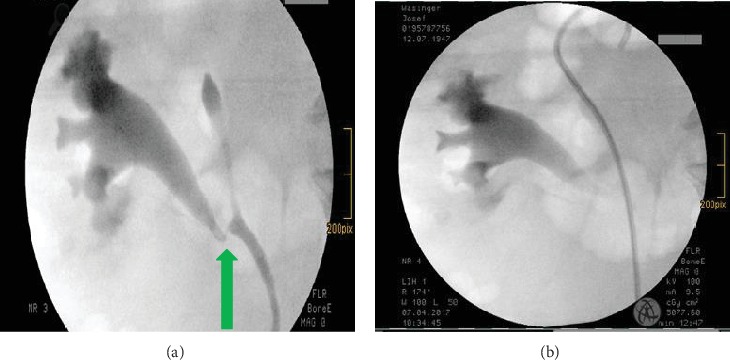
(a) Ureteral stricture on retrograde pyelogram. (b) Insertion of a double-J stent in the upper part of the duplex kidney.

A retrograde pyelography was performed, showing hydronephrosis of the upper part of the kidney and a jet phenomenon into the lower part, which was also dilated (Figure 2(a)). Semirigid ureteroscopy, using a 8/9.8-French ureteroscope (Wolf, Germany), failed due to a stenosis caused by scar tissue, but a guidewire could be placed in the upper part of the duplex kidney, and consecutively, a 7-French ureteral stent was placed via this guidewire (Figure 2(b)).

As the placement of the ureteral stent did not result in a decline of serum creatinine, a 10-French nephrostomy tube was additionally placed under ultrasound guidance in the dilated lower part of the duplex kidney.

After drainage of both collecting systems of the duplex kidney, serum creatinine levels decreased to 1.9 mg/dl and remained stable during follow-up.

**Figure 3:**
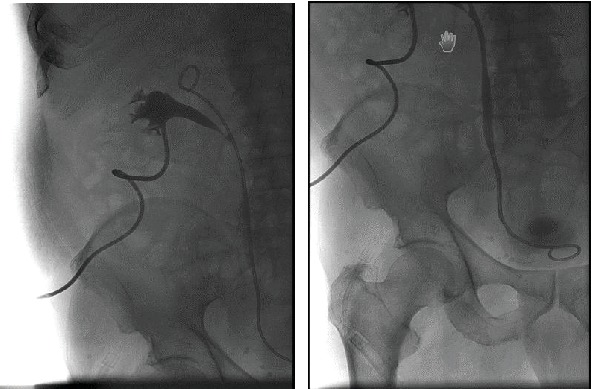
Antegrade nephrostogram with undisturbed contrast medium outflow through the lower kidney cavity system with the double-J stent still in situ.

One week after the second procedure, an antegrade nephrostogram was performed, which showed an undisturbed contrast agent discharge into the urinary bladder (with the double-J stent still in place) (Figure 3). Consequently, the nephrostomy tube was removed. The double-J stent was removed one month later.

Follow-up magnetic resonance imaging showed no RCC recurrence two years after RFA, the upper part of the duplex kidney was still dilated, the lower part showed no hydronephrosis with stable creatinine level at 1.9 mg/dl, and the patient remained asymptomatic.

## 3. Case Report Two

**Figure 4:**
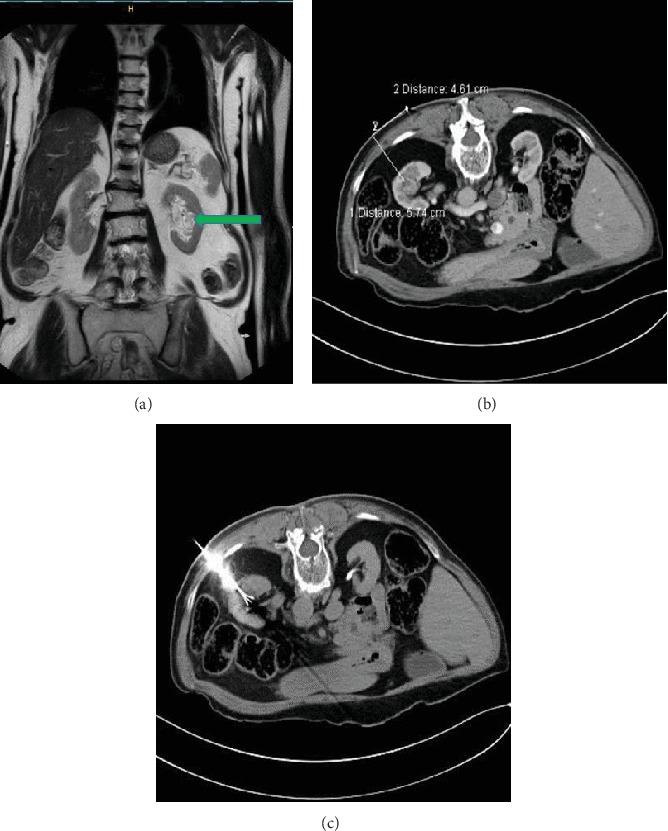
(a) 2 cm centrally located tumor of the left kidney before percutaneous RFA. (b) Planning the optimal ablation needle insertion path. (c) Needle with fully extended tine electrodes placed in the tumor.

Four months after an uncomplicated percutaneous CT-guided RFA (at the request of the patient and performed in the same fashion as described in case 1) of a two-centimeter, biopsy-proven centrally located papillary RCC in the middle third of the left kidney (Figures 4(a)–4(c)), a 78-year-old female patient was sent to our hospital by her urologist because of a grade II hydronephrosis of the upper calyces of the left kidney. She was asymptomatic, but creatinine had increased from 1.2 mg/dl before RFA to 1.9 mg/dl. CT scan showed neither signs of tumor recurrence nor urolithiasis.

**Figure 5:**
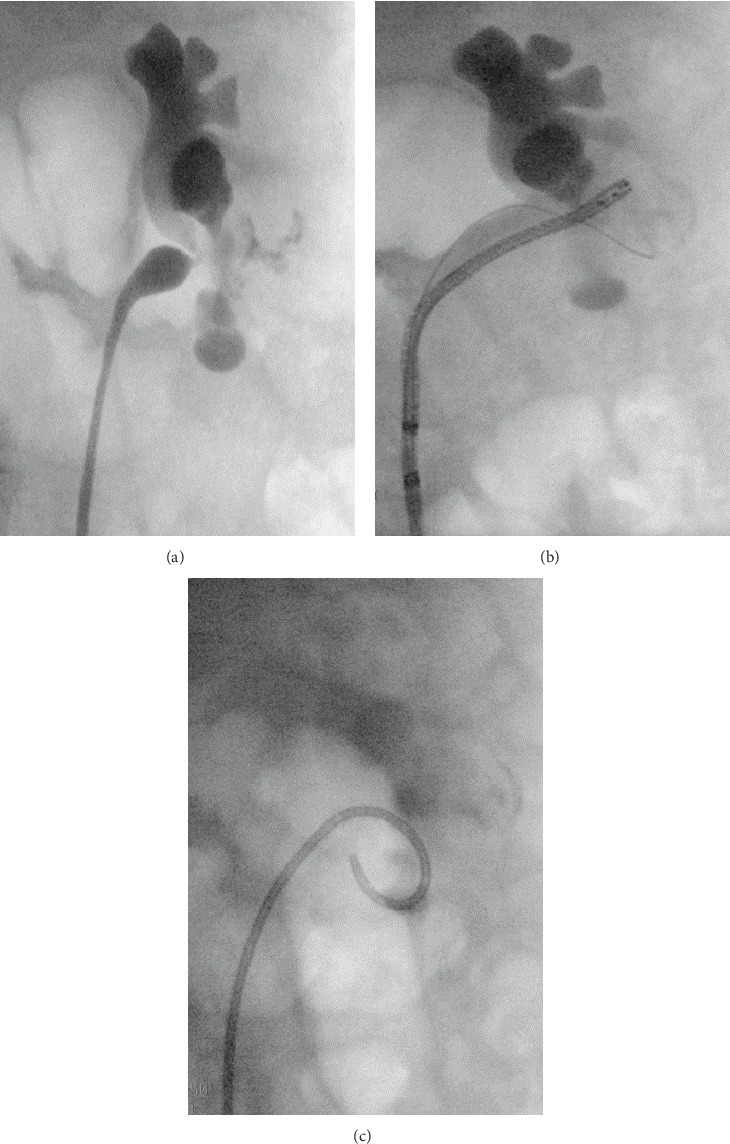
(a) Filiform stenosis at the pyeloureteral junction on retrograde pyelogram. (b) Approach of the middle calyx with flexible ureterorenoscope. (c) Insertion of a double-J stent in the lower calyx.

**Figure 6:**
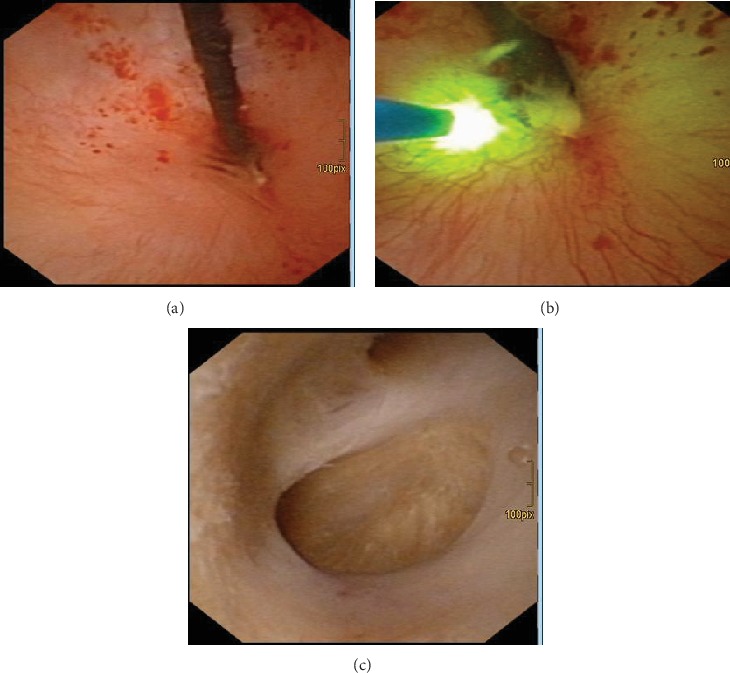
(a) Filiform stenosis at the pyeloureteral junction on flexible ureterorenoscopy. (b) Working up the stricture with a Vela XL Thulium-YAG Laser using a 230-micrometer laserfibre. (c) Access to the middle calyces with the flexible ureterorenoscope.

A filiform stenosis at the pyeloureteral junction was visible on retrograde pyelography (Figures 5(a) and 6(a)), which could be passed with a 0.035-inch guidewire. Under visual control using a flexible 9.9 F video ureterorenoscope (Olympus, Tokyo, Japan), the stricture was partially vaporized with a Vela XL Thulium-YAG Laser (Boston Scientific, Massachusetts, USA), using a 230-micrometer laserfibre at 25 watts (Figure 6(b)). The lower and middle calyces could be accessed (Figures 5(b) and 6(c)), and an ureteral stent was placed in the lower calyx (Figure 5(c)). The postinterventional creatinine decreased to 1.7 mg/dl, and getting access to the upper calyces is planned in a subsequent procedure, utilizing the same approach and technique.

## 4. Discussion

Renal cell carcinoma (RCC) is the most common cancer of the kidney and represents 2-3% of all cancers [4]. Small renal masses (SRM) < 4 cm (T1a) are increasingly found incidentally because of the widespread use of diagnostic abdominal imaging, such as ultrasonography, computed tomography, or magnetic resonance tomography for evaluation of other abdominal conditions, and now account for the majority of renal tumors [5, 6]. Surgery is the method of choice for localized RCC; however, there is a significant number of patients who are not ideal candidates for surgery because of comorbidities, chronic kidney diseases, multiple tumors, and anatomical or functional solitary kidney. RFA is emerging as a safe and effective alternative for these patients [1].

Minor complication are observed in 15-20%, e.g., postinterventional pain, fever, or hemorrhage, which resolve with conservative management [7]. Major complications that require interventions, including pneumothorax, injury to the bowel and the renal pelvis, or ureteral strictures, are rare. In a retrospective analysis of 98 percutaneous RFA performed at our institution from 2006 to 2016, no postinterventional stricture was observed [8]. To date, a total of 135 percutaneous RFA have been carried out at our institution, and the two described strictures result in a stricture rate of 1.5%.

Noncentral, posterior, and posterolateral tumors are easier to access and treat with low complication rates, whereas inferior, anterior, and medially located tumors bear higher chance of adjacent organ damage [9].

The “ABLATE algorithm” advocates placement of ureteral stent before ablation to avoid thermal-induced damage to the ureter, collecting system, or bowel. Thus, retrograde pyeloperfusion during the ablation may result in a conductive thermal “sink,” thereby protecting the ureter. If a renal tumor is 1 cm or less from the proximal ureter, an externalized 5- to 7-French ureteral stent is placed before the procedure and irrigated with sterile fluid during the ablation. The infusate flows retrograde through the stent into the renal pelvis and then returns antegrade within the ureter along the outside of the stent. The bladder is drained via a conventional bladder catheter. An additional advantage of having a ureteral stent in place is that it provides confident visualization of the ureter throughout the ablation procedure. If the tumor is 1 cm or less from the colon or small bowel, maneuvers to minimize the risk of bowel injury include simple changes in patient position (e.g., rolling the patient) and injection of either gas (pneumodisplacement using carbon dioxide) or fluid (hydrodisplacement with 5% dextrose in water) between the tumor and bowel for mechanical displacement [10] (Table 1).

Strictures of the collecting system and the ureter often develop over time and may not be present on the first postinterventional imaging; therefore, in the course of follow-up, special attention should be paid even in asymptomatic patients.

It has to be taken into account that patients who undergo RFA are not the best candidates for major open reconstructive surgery, so if strictures occur, they might be best managed by balloon dilation, endoureterotomy, and/or ureteral stenting, respectively.

## 5. Conclusion

Thermal injury-induced ureteral strictures may occur after RFA treatment if the ablated lesion is in close proximity to the ureter. The “ABLATE algorithm” may be helpful in reducing complication rates. Endoscopic management of ureteral strictures may be successful and necessary to preserve renal function in the affected kidney.

## Figures and Tables

**Table 1:** “ABLATE” algorithm [10].

ABLATE teaching points
A (axial tumor diameter)
Local treatment failures increase with increasing tumor size.
Ablation-related bleeding complications increase with increasing tumor size
If the tumor is ≥3 cm in diameter, consider cryoablation.
If the tumor is ≥5 cm in diameter, consider preablation tumor embolization.
B (bowel proximity)
Ablation-related bowel injury may result in long-term catheter drainage or surgery.
If the tumor is ≤1 cm from the colon or small bowel, patient repositioning or bowel displacement maneuvers will likely be necessary.
L (location within kidney)
Ablation can be performed safely and effectively in locations other than just the posterior and lateral kidney.
If the tumor is in the anterior kidney, hydrodisplacement will likely be necessary to protect adjacent bowel.
If the tumor is in the anterolateral upper pole of the right kidney, a transhepatic approach may be necessary.
If the tumor is in the anteromedial upper pole of the kidney near the adrenal gland, close blood pressure monitoring and even preablation *α*-receptor blockade may be necessary.
If the tumor is in the medial lower pole of the kidney, displacement techniques may be required to protect the nerves that run along the anterior surface of the psoas muscle.
A (adjacency to ureter)
Ablation-related ureteral injuries may require long-term stenting or surgery.
If the tumor is ≤1 cm from the ureter, retrograde pyeloperfusion via an externalized ureteral stent or ureteral displacement maneuvers will likely be necessary.
T (touching renal sinus fat)
Local treatment failures are more common with treatment of central tumors (those that touch renal sinus fat).
Ablation-related renal collecting system injuries and major bleeding complications are more frequent with treatment of tumors that touch renal sinus fat.
If the tumor touches renal sinus fat, consider cryoablation.
E (endo/exophytic)
Local treatment failures are more common with treatment of endophytic tumors (those that are completely contained within the renal capsule)
If the tumor is completely endophytic, consider ultrasound guidance, fusion guidance, or IV administration of contrast agent immediately before ablation for better lesion localization.

## Abbreviations

RCC: Renal cell carcinoma
CT: Computed tomography
RFA: Radiofrequency ablation
fURS: Flexible ureterorenoscopy.
